# Reconciling specificity and non-specificity in antibody binding: an energy landscape framework for immunology education

**DOI:** 10.3389/fimmu.2025.1650722

**Published:** 2025-09-04

**Authors:** Zhiyong Wang, Jiaqi Li, Min Wang, Yan Yu, Yanxin Lu, Qiang Xia, Pei Wei

**Affiliations:** ^1^ Department of Immunology, Zunyi Medical University, Zhuhai, China; ^2^ Department of Pharmaceutics, Zunyi Medical University, Zhuhai, China

**Keywords:** energy landscape, antigen, antibody, specificity, non-specificity

## Introduction

In the immune system’s toolkit for identifying foreign antigens, antibodies serve as pivotal molecular agents (1). An antibody’s function in immune recognition depends on its variable regions. These regions contain the key complementarity-determining regions (CDRs), which together create the antigen-binding site (the paratope) (2). The paratope, in turn, is designed to bind a specific 3D structure on an antigen, known as the epitope (3, 4). This interaction is complex, which is why immunology teaching has often relied on simplifying analogies. For a long time, the “lock-and-key” model was the standard, portraying specificity as a matter of a rigid, perfect structural fit (5, 6). But that view is too simplistic. The model was necessarily updated to the “induced-fit” concept, which accounts for the conformational flexibility of both molecules—they actively adjust to each other upon binding (7, 8). This evolution in thought makes it clear that molecular recognition is not a static event but a dynamic process, one that hinges on the structural adaptability of both partners.

The “lock and key” model and the “induced-fit” model have undeniably formed valuable ground on which to introduce molecular complementarity. This logical evolution is, indeed, quite extraordinary. However, when used to explain the vast complexity of immune recognition, these two models reveal the limits of that binary logic on which they are based: structural complementarity. Whether the interaction is static or dynamic, the core evaluative question remains binary: do the molecules “fit” or not? This same assumption creates an ongoing pedagogical conundrum, one which cannot be resolved through mere refinements of the existing models. On the one side, students are taught that antibodies have “absolute specificity”, strongly binding only to that single, well-defined target molecule. On the other side, this scenario stands in stark contrast to established immunological phenomena such as cross-reactivity-given where a single antibody binds to structurally distinct antigens or where broad polyreactivity is observed in natural antibodies (9–12), for example, natural IgM (13–15). Within a framework governed by an all-or-nothing notion of molecular matching, these realities appear contradictory and difficult to reconcile.

This fundamental paradox gives rise to two immediate and significant consequences. First, at the pedagogical level, it imposes a considerable cognitive burden on students, who are expected to reconcile two seemingly contradictory concepts: “specificity” and “non-specificity” (polyreactivity). This often leads to confusion and fragmented understanding. Second, at the conceptual level, the binary framework promotes a tendency to dismiss nonspecific interactions as “meaningless”, “erroneous”, or merely “background noise”. This viewpoint neglects the fundamental physiology in which weak interactions participate in critical biological processes of immune surveillance, alteration of T-cell activation thresholds, and fine-tuning of signaling pathways (13–16). Hence, there is a compelling need for more integrative theoretical models that can reconcile the apparent contradictions between specificity and non-specificity as well as between high- and low-affinity interactions when teaching antigen-antibody binding.

## Energy landscape theory: a unified physical framework of “specific” and “nonspecific” binding

**Figure 1:**
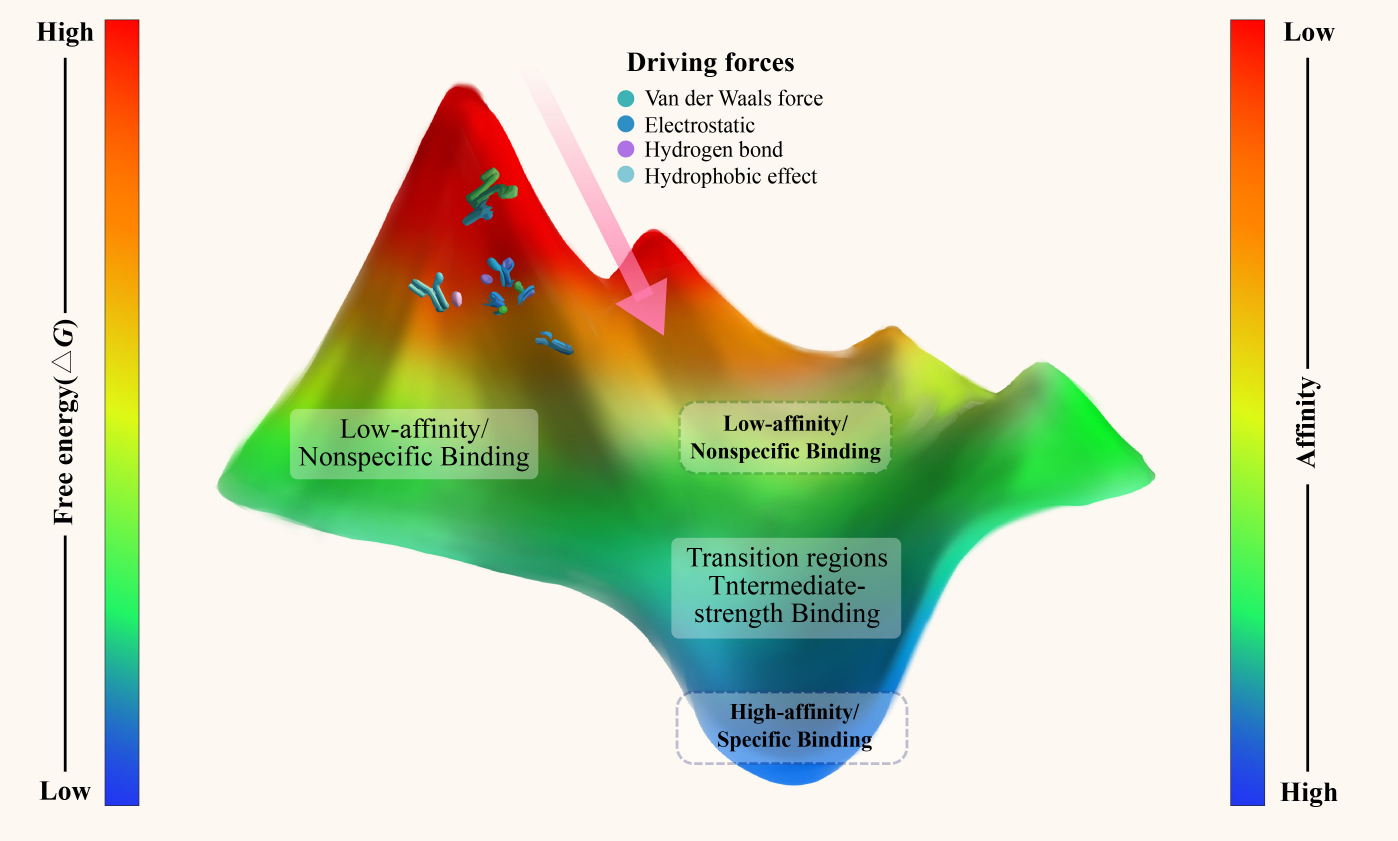
Energy landscape of antigen-antibody binding. This schematic represents the antigen-antibody binding process as a continuous energy landscape, where free energy (Δ*G*) varies from high (red) to low (blue) values, corresponding to binding affinities from weak to strong. Deep energy wells indicate high-affinity, specific interactions characterized by tight structural complementarity and strong noncovalent forces, including hydrophobic, electrostatic, hydrogen bonding, and van der Waals interactions. In contrast, shallow wells represent low-affinity, non-specific interactions formed through more generic molecular interfaces. The optimal binding process is depicted as a descent from higher to lower energy states. Affinity maturation is illustrated as the sculpting of deeper, more defined wells, enhancing specificity while preserving shallow interactions for broad-spectrum immune surveillance.

A few refinements on old concepts alone will not help us to transcend the limits of classical structural models. What is called for is an entirely new conceptualization that draws upon existing insights but provides a greater explanatory foundation. At the microscopic scale of antigens and antibodies, these entities resemble dynamic particles whose movements and interactions can be understood in accordance with the physical science laws of nature, including thermodynamics and statistical mechanics. Thus, the energy landscape theory-imported from the field of physics-may be the grand unifying theory for molecular recognition in immunology. The binding of antibodies to antigens and unbinding are energy transitions on the energy landscape, where molecular conformations follow successive pathways toward thermodynamically favorable states. From a thermodynamic view point, Gibbs free energy change (Δ*G*) is the primary variable used to characterize the quantitative nature of molecular binding events (17, 18). At constant temperature and pressure, a negative Δ*G* indicates that the reaction is spontaneous with a tendency to occur, while a positive ΔG indicates that the reaction is nonspontaneous and may not occur (17, 18). The energy landscape itself can be thought of as a topological map quite intuitively, where “altitude” at a given point represents the free energy associated with a certain molecular conformation (**Figure 1**). Antigen-antibody binding under this scheme is an altogether different “fit”-it is a dynamic process in which the system is exploring the energetic terrain and stochastically settling into lower-energy regions, known as energy wells.

In this framework, high-affinity interactions are represented as deep and sharply defined energy wells. These binding events are typically characterized by a Δ*G* ranging from approximately -7 to -14 kcal/mol, which thermodynamically drives spontaneous molecular association (19, 20). This substantial free energy decrease arises from a delicate balance of enthalpic and entropic contributions, as captured in the thermodynamic equation Δ*G* = Δ*H* - TΔ*S* (21, 22). A large negative enthalpy change (Δ*H*), often due to the precise geometric complementarity at the antibody-antigen interface (23), allows extensive non-covalent interactions such as hydrogen bonding, van der Waals forces, hydrophobic packing, and electrostatic interactions (24–27). At a molecular level, specific amino acid residues play key roles in this enthalpic drive. Tyrosine (Tyr) and tryptophan (Trp) contribute substantially through direct bonding and electrostatic interactions, while arginine (Arg) and aspartic acid (Asp) often form stabilizing salt bridges (28–31). A critical aspect of this process is the conformational preorganization of mature antibody binding sites (32, 33). These sites adopt a binding-competent conformation prior to antigen engagement (34), thereby minimizing the entropic penalty associated with structural ordering upon binding (i.e., reducing the unfavorable -TΔ*S* term). In fact, affinity maturation in the immune system sculpts the energy landscape through somatic hypermutation, progressively refining the binding site to deepen and narrow the energy well (35–37). This consolidation of local minima into a single global minimum leads to significantly slower dissociation rates (k__off_) and prolonged antigen-antibody residence times (38–40).

In stark contrast, lower-affinity or “non-specific” binding corresponds to the presence of broad, shallow energy basins on the molecular energy landscape. These interactions arise from more generic, less structurally refined molecular interfaces (31, 41). Importantly, such interactions are not errors or random noise; rather, they reflect a functional mode of recognition. In regions where precise geometric and chemical complementarity is lacking, fewer stabilizing interactions, such as hydrogen bonds and electrostatic contacts, are formed, leading to a less stable antigen-antibody complex (31, 41). Kinetically, this is reflected in the dynamic behavior of the antibody molecule itself. The CDR loops exhibit local conformational fluctuations on the picosecond-to-nanosecond timescale, while larger structural rearrangements occur over microseconds (42, 43). This continuous conformational sampling enables a single antibody to engage transiently with multiple, structurally diverse antigens. As a result, these interactions are characterized by rapid dissociation rates (k__off_ typically ranging from 10⁻¹ to 10¹ s⁻¹), yielding short residence times on the order of milliseconds to seconds (44, 45). Biologically, such transient interactions are far from inconsequential. Natural IgM exemplifies this polyspecific behavior: despite having relatively low affinity at individual binding sites, their pentameric structure provides strong overall avidity (46, 47). This architectural advantage allows the immune system to prioritize breadth over precision, facilitating rapid, high-throughput scanning of the molecular environment. In doing so, it establishes a critical first layer of immune surveillance, capable of detecting a wide array of potential threats with minimal prior information (13–15).

Therefore, the energy landscape theory redefines molecular binding as a probabilistic event, effectively bridging the perceived divide between “specific” and “non-specific” interactions by placing them along a continuous spectrum. These categories are no longer seen as fundamentally distinct, but rather as different outcomes governed by the same underlying physical principles. The distinction shifts from a binary, yes-or-no assessment to one based on probability and residence time. Specifically, the likelihood that an interaction will occur (determined by the depth of the energy well, Δ*G*), and the duration for which it persists (inversely related to the dissociation rate, k__off_). Within this framework, interactions ranging from high-probability, long-residence “specific” bindings to low-probability, short-lived “cross-reactive” events are unified under a single conceptual model. This shift enables a more integrated understanding of antibody function, connecting molecular-scale physicochemical properties with immune system behavior at the systems level. It lays the groundwork for a comprehensive theoretical foundation that can better explain both the precision and flexibility of immune recognition.

## Implications of the energy landscape theory for immunology education

Integrating energy landscape theory into immunology education is a pedagogical paradigm shift, not merely a conceptual update. It requires a transition from static or singularly dynamic structural descriptions to a broader understanding of dynamic processes, and from absolute specificity perception to relative interaction probability assessment. With this shift, students can have a more profound understanding of the essence of antibody recognition; new approaches can also be developed to resolve difficulties encountered in traditional teaching.

Energy landscape diagrams can be used to explain cross-reactivity and polyspecificity in a way that is inaccessible to both the lock-and-key and induced-fit models. They can be used to show differences in affinities when an antibody binds to different antigens by using diagrams that show deep wells for target antigens and shallower wells for low-affinity, cross-reactive antigens. They show that cross-reactive, low-affinity antigens only enter shallow wells with tertiary states only relevant to molecular structure and affinity differences. Using structural data, students can see examples of binding interfaces, where they can analyze adhesion in terms of the size of contact area, number of bonds, and hydrophobic interactions that enable affinity differences to be connected to xenobiotic molecular structures. They should also determine how cross-reactivity in immunity plays critical and essential roles, including broadly conferred antiviral immunity and maintenance of autoimmune tolerance. Polyreactive antibodies have unique characteristics that facilitate broad recognition because of the low-affinity and wide range of different binding capabilities that can be represented by shallower energy wells. Therefore, polyreactive antibodies may be produced quickly to respond to immune responses through surveillance mechanisms.

Furthermore, the energy landscape framework provides a powerful lens through which to understand the very first step of adaptive immunity: the selection of naive B cells for activation. Before affinity maturation can even begin, a naive B cell must be chosen from a vast repertoire based on its initial interaction with an antigen. This selection process can be conceptualized as a binding energy threshold. For a B cell to be activated, the binding of its B-cell receptor (BCR) to an antigen must be stable enough—that is, it must fall into an energy well of sufficient depth—to generate a sustained intracellular signal that surpasses this activation threshold. Interactions that are too transient (in very shallow energy wells) will fail to trigger a response. Thus, the energy landscape not only describes the subsequent optimization process within the germinal center but also governs the initial “go-or-no-go” decision that determines which B cells are deemed worthy of entering the affinity maturation pathway. This helps students connect the abstract concept of binding energy directly to the concrete biological outcome of cellular selection.

When explaining the mechanisms of affinity maturation, somatic hypermutation can be linked to energy landscape optimization. By showing students the antibody gene mutation selection process during affinity maturation, they can observe how antibodies gradually “adjust the shape of the key” or, more accurately, reshape their energy landscape to find the optimal conformation for antigen binding, ultimately “sliding down” to the lowest point of the energy landscape. Computational simulation tools can be used to demonstrate how antibody mutations alter the binding free energy, deepening and narrowing energy wells, consequently enhancing antibody affinity and specificity.

When explaining antibody effector functions, binding kinetics can be linked to functional regulation. The way by which the stability of antigen–antibody binding affects downstream effector functions (complement activation, antibody-dependent cell-mediated cytotoxicity) can be illustrated by introducing kinetic parameters such as k__off_ and residence time. Students can be guided to consider how to engineer antibodies to optimize their binding kinetics, thereby improving their therapeutic efficacy.

Ultimately, we should focus on the roles of nonspecific binding in the immune system (including, but not limited to, immune surveillance, immune cell migration, and regulation of the inflammatory response). We should also encourage students to explore the “dark matter” of nonspecific binding and determine whether they could possibly exploit nonspecific interactions in new immunotherapy applications. We should discuss polyreactive antibodies as a key aspect of nonspecific binding and their connection to these larger aspects of immune function.

In conclusion, the energy landscape theory should be incorporated into immunology education to help students develop a more advanced understanding of antibody recognition and enhance their scientific and creative thinking. By focusing on the full binding affinity spectrum of universality, more specifically including the functional importance of nonspecific interactions and polyreactivity, students can develop a more complete and deeper understanding of immune recognition.

## Challenges of applying the energy landscape theory in immunology teaching and responses

The energy landscape theory is a new lens through which we can revisit antigen–antibody recognition and interactions between immune cells. It is based on the idea about a potential energy surface in physics; it also allows the visualization of molecular interactions as topographic maps of energetic changes, thereby clarifying features such as binding affinity, kinetics, and specificity. However, similar to other types of interdisciplinary teaching, adopting this theory in immunology classrooms has challenges, requiring attentiveness and responsivity to teaching practice.

The main hurdle is the cognitive barriers represented by the abstract nature of the concept. Energy landscapes, which are an abstract concept, depict principles from thermodynamics and statistical mechanics that can be difficult for students. Students may easily experience a disconnect between abstract energy curves and actual molecular interactions. Hence, teaching engineers and scientists should lower the theoretical wall by using understandable language, stimulating analogies, and linking abstract ideas as much as possible to everyday experiences. Importantly, if the visual teaching package is well developed such that students can experience and co-construct the notion of energy landscape for biologically relevant systems through computer simulation, animation, or interactive VR models, then students can learn these notions as dynamic and intuitive processes. Most of them will tend to implicitly understand model systems describing the fundamental concepts of binding affinity and the dynamic rates of association and dissociation of association.

Beyond in-class pedagogical strategies, a more structural approach at the curricular level could also be highly effective. For instance, institutions could consider offering an introductory short course or module in physical biochemistry as a prerequisite or co-requisite for the immunology course. Such a preparatory course would equip students with the foundational concepts of thermodynamics, kinetics, and the energy landscape itself, thereby significantly lowering the cognitive barrier when these ideas are applied to the complex context of immunology. This would create a smoother and more integrated learning experience for students.

Second, the energy landscape model can simplify complex biological systems; however, the pure and simple use of this model can cause students to become unaware of the intricate factors (e.g., solvent effects, molecular crowding, and glycosylation modifications) present in actual biological environments. As such, immunology education should not only describe the energy landscapes but also highlight the interaction events on antigen–antibody complexes that happen in real biological environments, where many factors influence these interactions. Teachers can use case study discussions to urge students to consider the factors that create challenges for antigen–antibody binding; thus, they help enhance the systemic thinking of students.

Another issue that needs attention is cognitive dissonance caused by conflicts with traditional teaching content. Energy landscape and traditional immunology theories, such as the “lock-and-key” and “induced-fit” models, have different descriptions of molecular recognition mechanisms, which may cause cognitive conflict among students. Therefore, teachers explaining energy landscapes should avoid directly negating traditional models; instead, they should adopt a comparative analysis approach to highlight the advantages and limitations of each theory. They should also emphasize that the energy landscape theory is a supplement to and an improvement of traditional models rather than a replacement. For example, they can encourage students to consider the advantages of the “lock-and-key” model in explaining high-specificity binding and the utility of the “induced-fit” model in describing the conformational flexibility required for many interactions. Then, they can demonstrate how the energy landscape provides a unifying physical basis that explains why and how these phenomena occur, while also accounting for cross-reactivity and polyreactivity, which the other models cannot. They should specifically address potential students’ misconceptions about “nonspecific” (low-affinity) binding and emphasize the clarification of the energy landscape perspective on its important roles through polyreactive antibodies.

Teachers should prevent students from falling into the trap of reductionism. When applying the energy landscape theory to analyze antigen–antibody interactions, teachers risk simplifying complex biological processes into a superposition of intermolecular forces while neglecting the regulatory role of the immune system as a whole. Therefore, teachers should highlight the holistic nature of the immune system and the complex relationships between antigen–antibody interactions and other immune cells, cytokines, and other factors. Thus, teachers can incorporate typical immune disease cases to guide students in the analysis of immune system dysregulation in disease states and the role of antigen–antibody interactions in the occurrence and development of diseases.

In conclusion, incorporating the energy landscape theory into instructions on immunology may help expand students’ thinking and understanding of the meaning of antigen–antibody interactions. However, in practice, instructors should be aware of the challenges; furthermore, they may use the role of the energy landscape theory more effectively in immunology education through innovation in teaching methods, focusing and developing content, and increasing the link between theory and practice.

## Conclusion

The theory of energy landscapes will be proposed as a more fundamental physical model for resolving the classical teaching paradox in immunology-the impossibility of reconciling antibody specificity and cross-reactivity. The theory does not contest structural complementarity but relates it to a probabilistic and dynamic context, so that specific and non-specific interactions are taken under the continuum of energy and time. This should help students to construct a theoretical model of immune recognition that consists of affinity maturation, cross-reactivity, and the surveillance of immunity all in one phenomenon, ultimately cultivating a deeper, probabilistic scientific understanding.
